# Effects of whole-body vibration training in a cachectic C26 mouse model

**DOI:** 10.1038/s41598-021-98665-7

**Published:** 2021-11-03

**Authors:** Miranda van der Ende, Rogier L. C. Plas, Miriam van Dijk, Jvalini T. Dwarkasing, Frans van Gemerden, Attusa Sarokhani, Hans J. M. Swarts, Evert M. van Schothorst, Sander Grefte, Renger F. Witkamp, Klaske van Norren

**Affiliations:** 1 Division Human Nutrition and Health, Nutritional Biology and Health, Wageningen University & Research, Wageningen, The Netherlands; 2 Human and Animal Physiology, Wageningen University & Research, Wageningen, The Netherlands; 3 Nutricia Research, Nutricia Advanced Medical Nutrition, Utrecht, The Netherlands

**Keywords:** Muscle, Skeletal muscle, Cancer

## Abstract

Targeted exercise combined with nutritional and pharmacological strategies is commonly considered to be the most optimal strategy to reduce the development and progression of cachexia. For COPD patients, this multi-targeted treatment has shown beneficial effects. However, in many, physical activity is seriously hampered by frailty and fatigue. In the present study, effects of whole-body-vibration-training (WBV) were investigated, as potential alternative to active exercise, on body mass, muscle mass and function in tumour bearing mice. Twenty-four male CD2F1-mice (6–8 weeks, 21.5 ± 0.2 g) were stratified into four groups: control, control + WBV, C26 tumour-bearing, and C26 tumour-bearing + WBV. From day 1, whole-body-vibration was daily performed for 19 days (15 min, 45 Hz, 1.0 g acceleration). General outcome measures included body mass and composition, daily activity, blood analysis, assessments of muscle histology, function, and whole genome gene expression in m. soleus (SOL), m. extensor digitorum longus (EDL), and heart. Body mass, lean and fat mass and EDL mass were all lower in tumour bearing mice compared to controls. Except from improved contractility in SOL, no effects of vibration training were found on cachexia related general outcomes in control or tumour groups, as PCA analysis did not result in a distinction between corresponding groups. However, analysis of transcriptome data clearly revealed a distinction between tumour and trained tumour groups. WBV reduced the tumour-related effects on muscle gene expression in EDL, SOL and heart. Gene Set Enrichment Analysis showed that these effects were associated with attenuation of the upregulation of the proteasome pathway in SOL. These data suggest that WBV had minor effects on cachexia related general outcomes in the present experimental set-up, while muscle transcriptome showed changes associated with positive effects. This calls for follow-up studies applying longer treatment periods of WBV as component of a multiple-target intervention.

## Introduction

The cachexia syndrome, among others, characterised by disease-induced loss of muscle mass, can occur in patients with chronic kidney disease, heart failure, Chronic Obstructive Pulmonary Disease (COPD) and cancer^1,2^. It is associated with reduced treatment efficacy and a reduced quality of life^3–6^. Although the symptoms of this syndrome show a high degree of similarity for different diseases, the causes are multiple and their contribution to the development of cachexia may differ per disease and treatment trajectory^1,7^. Moreover, it has been shown that cross-talk between different organs plays an important role, which makes cachexia a complex syndrome that is difficult to treat^8–10^. There is increasing consensus between experts that cachexia should be treated with a multimodal approach addressing dietary composition and intake, attenuating systemic inflammation, and stimulating physical activity^5,6^. For COPD, there is already an evidence-based treatment combination of exercise and dietary treatment^11–13^. For cancer patients, however, less data is available supporting such a treatment.

A systematic review of the effects of physical exercise on muscle mass and strength in cancer patients provided evidence that resistance training, aerobic training, or a combination of these can improve muscle strength^14^. However, coexisting fatigue and frailty are often limiting factors in these patients^15^. Therefore, there is a need for training methods that are easily accessible for frail patients. Vibration training might meet this demand, since it provides an easily accessible and low intensity type of exercise. Patients stand on a vibrating surface, which transmits the vibration from the plate to the patient^16^. In older individuals, positive effects of whole-body-vibration training (WBV) on VO_2,max_ and muscle strength have been observed^17^. Moreover, vibration training increased exercise capacity in patients treated for respiratory cancer^18^. Additionally, it was found that resistive vibration exercise could prevent the decrease in mitochondrial respiration during bed rest^19^. However, literature data are scarce, and studies are difficult to compare due to differences in set-up. A meta-analysis reported a beneficial effect on leg muscle strength in elderly^20^, whereas a systematic review concluded that there would be only weak proof of efficacy of WBV in elderly^21^. Due to the overall inconsistent results of WBV, it remains unclear whether WBV could be a beneficial intervention to improve muscle mass and function in elderly. However, with a recent Delphi consensus study a more consistent form of reporting WBV studies is at hand^22^. Moreover, WBV has never been thoroughly investigated during disease-driven net catabolic conditions. Therefore, in this study, the effects of WBV alone using a murine cancer cachexia model were investigated.

Effects of WBV in rodents found so far seem promising but have also provided contradicting results. For example, WBV showed to be beneficial in chronic pain, it reduced mechanical and thermal sensitivity in male Wistar rats^23^. In healthy mice, one study has shown that low-intensity WBV can partially improve muscle contractility, in particular strength and relaxation rates^24^. Although that WBV is having a positive effect in young mice, this effect could not be found in aged mice^25^. Another study demonstrated effectiveness of WBV in suppressing muscle atrophy pathways both in vivo and in vitro. Apart from effects on muscle, WBV is also able to improve bone health in mice and rats^26,27^. Investigating the effect of vibration training in a mouse model for Duchenne muscular dystrophy showed no effect on bone or muscle improvement^28^. However, no studies were found in literature investigating a condition of cancer-induced atrophy, additionally no Delphi study is performed for animal WBV studies yet. So, the question whether WBV might be able to attenuate muscle wasting during this disease remains unknown.

To this end, the effects of WBV were investigated in a colon-derived-tumour-induced C26 cachexia mouse model, studying cachexia outcomes at different levels, including the muscle transcriptome, markers for bone and muscle function, muscle mitochondrial biogenesis, body composition and muscle performance. It is hypothesized that WBV might be able to attenuate muscle wasting in colon-derived-tumour-induced C26 cachexia mouse model.

## Materials and methods

### Tumour model

Twenty-four male CD2F1 mice weighing ~ 20 g (6–8 weeks, BALB/c x DBA/2, Charles River, The Netherlands) were individually housed in macrolon type 3 cages with sawdust and tissues as nesting material, in a climate-controlled room (21 °C ± 1 °C) with a 12:12 h dark–light cycle. Sample size was small because of the explorative nature of this study. Mice had ad libitum access to chow and water. Upon arrival, mice were stratified on body mass and divided into groups of 6 animals (Control [C], Control + whole body vibration training [C + WBV], Tumour bearing [T] and Tumour bearing + whole body vibration training [T + WBV]). Subsequently, mice were allowed to acclimatize for 1 week prior to the start of the experiment. Murine C26 adenocarcinoma cells, kindly obtained from the lab of Donna McCarthy (Ohio State University, USA), were cultured and suspended as described previously^29^. Tumour cells (1 × 10^6^ cells in 0.2 ml of Hanks’ balanced salt solution (HBSS)) were inoculated subcutaneously into the right inguinal flank of the mice under general anaesthesia (isoflurane/N_2_O/O_2_). HBSS was used as sham injection (0.2 ml). This study was conducted in accordance with institutional guidelines for the care and use of laboratory animals established by the Animal Ethics Committee of the University of Wageningen, and all animal procedures related to the purpose of the research were approved under the Ethical license of the national competent authority (registration number 2014075.e), securing full compliance the European Directive 2010/63/EU for the use of animals for scientific purposes and the ARRIVE guidelines.

### Experimental design

From day-5 (D-5) onwards body mass, activity and grip strength were measured daily. On day 0 (D0), an injection with tumour cells or HBSS was given and blood was collected using a tail-vein cut. On D0, D7, D14 and D19, body composition, i.e. lean mass and fat mass, was measured using an EchoMRI Whole Body Composition Analyzer (EchoMRI, Houston, TX, USA). Starting on D1, mice were subjected to a whole-body vibration training (WBV) protocol, similar to previous rodent studies^24,26,28,30^, with 15 min of vibration training for 7 days/week, with a frequency of 45 Hz and 1.0 g acceleration.

The vibration platform used was an adjusted commercially available VG^®^ Professional (VibroGym, Badhoevedorp, The Netherlands) power plate. Four Plexiglas cages were mounted onto the power plate to enable WBV in four mice simultaneously. The power plate was calibrated using accelerometers on all four corners to ensure the frequency and acceleration specifics. On D19, at least 24 h after the last training session and after anaesthesia, blood was collected by cardiac puncture and animals were killed. Subsequently, organs and hindlimb muscles were weighted and snap-frozen in liquid nitrogen. Carcass mass was determined as body mass excluding tumour weight. *M. soleus* (SOL) and *m. extensor digitorum longus* (EDL) were divided in two parts: one for histology and one for RNA isolation. The EDL and SOL of the other leg were used for ex vivo muscle function. Front legs were frozen for bone mineral density (BMD) measurement.

To assure compliance of mice with the WBV, a small pilot was executed prior to the experiment. Three additional control mice were used and apart from the vibration training no other treatment was applied. Mice did not show any signs of aberrant behaviour when exposed to the vibration training. Video recordings of mice subjected to WBV showing the behavioural response to the training are provided in supplemental video 1.

### Grip strength

Forelimb grip strength was measured daily as previously described^31^, using a calibrated grip strength apparatus from Panlab (Cornella, Spain), following the protocol delivered with the equipment. Each day, a set of five maximum effort repetitions was performed. From these five measurements, the average of the middle three measurements was determined. To eliminate measurement variation, grip strength was expressed per mouse as mean grip strength on D15-18 as a percentage of the mean on D-3 to D0.

### Daily activity

Physical activity was monitored throughout the acclimatisation and study period starting at D-7, using activity sensors (dual technology detector DUO 240, Visonic; adapted by R Visser, NIN, Amsterdam, The Netherlands) according to an adapted protocol previously described^32^. Sensors translated individual disruption of the infrared beams caused by movements of the animals into arbitrary activity counts. Sensors were mounted above the home cages and connected through input ports and interface to a computer equipped with MED-PC IV software for data collection (MED associates, St Albans, VT, USA). Activity was expressed in counts per half hour. The activities of each 12 h dark cycle were summed to dampen the hour-to-hour variability. To eliminate measurement variation, daily activity was expressed per mouse as mean daily activity on D16-19 as a percentage of the mean on D-3 to D0.

### Bone mineral density and DEXA

Frozen front legs were used for determination of BMD measured by dual energy X-ray absorptiometry (DEXA) scan, using a PIXImus imager (GE Lunar, Madison, WI, USA).

### Blood plasma cytokines and PTHrP

The following cytokines were measured using a mouse cytokine Milliplex bead immunoassay (MCYTOMAG-70K-09, Merck chemicals^33^, Amsterdam, The Netherlands): IFNγ, IL1-β, IL4, IL6, IL10, IL15, MCP1, TNFα and VEGF. PTHrP was measured using a quantitative PTHrP enzyme-linked immunosorbent (ELISA) assay kit (SEA819Mu, Cloud-Clone Corp., Uscn Life Science Inc., Wuhan, Hubei, PRC).

### Ex vivo muscle function

At the end of the experiment (D19), ex-vivo muscle function of right SOL and EDL was measured according to an adapted protocol previously described^34^. Muscles stabilized in the organ bath for 30 min. Subsequently, optimal stimulation strength was determined. Force frequency characteristics (10–167 Hz, 250 ms (EDL) and 500 ms (SOL)) were determined after refreshing the organ buffer and 5 min of rest. Next, an exercise protocol was performed of 100 contractions (83 Hz, 250 ms every 1000 ms for EDL and 83 Hz, 500 ms every 2000 ms for SOL). At the used frequencies, complete tetanic contraction of the muscle was reached. Force signals of the force frequency curve and the exercise protocol were analysed for maximal force (F_max_), contraction and relaxation time (CT and RT respectively) and rate of change of force (dF/dt). Area under the curve (AUC) for F_max_ was determined for both force frequency and exercise protocols. CT, RT and dF/dt were analysed between 83 and 167 Hz.

### Immunofluorescent histology

#### Staining MHC type I in m. soleus

Immunofluorescent staining was performed as previously described^35^. SOL sections (10 µm) were cut using a cryostat at –20 °C, air dried and stored at − 20 °C until use. Based on MHC isoform, fibre-type abundancy was determined and classified as MHC type I and II using monoclonal antibody against mouse MHC type I (BAD-5, 1 μg/mL, as developed by Schiaffino; the Developmental Studies Hybridoma Bank, The University of Iowa, IA, USA) and immunofluorescent secondary anti-mouse immunoglobulin G2b antibody (IgG2b Alexa F488, 2 μg/mL; Fisher Scientific, Landsmeer, The Netherlands). Normal goat serum was diluted 1:10 (dNGS) in phosphate buffered saline (PBS). Sections were first air-dried for 15 min and then blocked with dNGS for 60 min at 37 °C. Primary antibody dilution was prepared in dNGS, added to the sections and incubated for 60 min at 37 °C. Sections were gently washed three times with PBS. Secondary antibody dilution was prepared in dNGS and added to the slides to incubate for 60 min at 37 °C in the dark. After incubation, sections were gently washed again three times with PBS. Wheat germ agglutinin dilution (WGA, 20 μg/mL) was prepared in PBS. WGA was added to the sections and incubated for 20 min at 37 °C in the dark. Sections were gently washed three times PBS before enclosure with Vectashield-hard set (with DAPI; Fisher Scientific).

#### Fibre area analysis

Gray-scale images of the sections were taken under a Leica DMIL LED microscope with a 10 × objective (Leica Microsystems, Amsterdam, The Netherlands). Images were taken at 10 × magnification. The microscope was equipped with Red (Excitation: BP 546/11 nm; Emission BP 605/75 nm), Green (Excitation: BP 470/40 nm; Emission BP 525/50 nm), and Blue (Excitation: BP 360/40 nm; Emission LP425 nm) filters, a Leica DFC450C camera (resolution of 52294 DPI), and LAS X 2.0 software (Leica). Images were combined using ImageJ 1.15f for Windows (National Institutes of Health, Bethesda). Total area of a muscle section was analysed using ImageJ. Muscle fibre properties were analysed using SMASH muscle image analysis application for MATLAB r2015b (Mathworks, Natick)^36^. Abundance of type II muscle fibres was measured as MHC type I negative fibres as percentage of total fibre count. Muscle fibre cross-sectional area (CSA) was determined by measuring minimal Feret’s diameter of fibres, a reliable measure for CSA^37^. The ratio of type II/type I fibre CSA and the relative abundance of type II MHC (total type II CSA as % of total muscle CSA) were also determined.

#### qPCR gene expression

RNA was isolated from gastrocnemius skeletal muscle tissue. Frozen tissue was added to ice-cold Trizol Reagent (Invitrogen Cat. No. 155596-026) and homogenised for 60 s using the IKA Ultra-Turrax T25 homogenizer. After centrifugation chloroform was added. Samples were centrifuged again, and 2-propanol was added to the aqueous phase. The supernatant was removed after centrifuging, and the remaining pellet was washed once in 75% ethanol by vortexing, followed by centrifuging. Finally, the pellet was air-dried and resuspended in nuclease-free water by an incubation at 55 °C in a heating block. The RNA concentration and purity were assessed with the Nanodrop spectrophotometer (Nanodrop ND1000, Nanodrop technologies Wilmington, DE, USA). cDNA was synthesized using the Promega cDNA synthesis Kit (A3500, Promega Benelux B.V., Leiden, the Netherlands) following the manufacture’s instruction in a Hybaid PCR machine. qPCR was carried out using Sensi-Mix SYBR-green. A threefold dilution series consisting of six dilutions were prepared for the standard curve. Primer sets for *Ampk* (AMP-activated protein kinase), *Pgc1α* (*Ppargc1a*, Peroxisome proliferator-activated receptor-γ coactivator 1 alpha), *Sirt1* (silent mating type information regulation 2 homolog 1), *Akt1*, *Atrogin1* (*Fbxo32*), *Murf1* (*Trim63*), *β-actin* and *Tbp* (TATA-sequence binding protein) were used (table S1). *β-actin* and *Tbp* were used as reference genes in order to normalize the data for the target genes. Relative fold change was calculated with the 2-ΔΔCt method as described previously^38^ and in the User Bulletin #2 (ABI PRISM 7700 Sequence Detection System) of Applied Biosystems.

#### Protein expression

Total protein was extracted from frozen gastrocnemius skeletal muscle tissue by homogenising the tissue in RIPA buffer (50 mM Tris–HCl pH 7.4, 150 mM NaCl, 1 mM EDTA, 1% NP-40, 0.25% Na-deoxycholate, 10 × phosphatase inhibitor (Roche Diagnostics) and 25 × Protease inhibitor (EDTA complete)) using an Ultra-Turrax homogeniser. The protein concentration was quantified with the BCA protein assay (Pierce) according to the manufacturer’s directions. Equal amounts of each protein sample (20 μg) were loaded on an 8–16% SDS gel (Biorad) and separated by SDS-PAGE. The separated proteins were transferred on a PVDF membrane followed by a 1 h blocking step in 5% (w/v) milk. Membranes were incubated with different primary antibodies AKT1 (1:1000, Cell Signaling, #9272S), p-AktSer473 (1:2000, Cell Signaling, #9271S), AMPKa (1:1000, Cell Signaling, #2532S), p-AMPKaThr172 (1:1000, Cell Signaling, #2535S), Ppargc1aα (1:1000, Invitrogen, PA5-72948) and p-hPpargc1a Ser571 (1:500, R&D Systems, AF6650), SIRT1 (1:1000, Cell Signaling, #2493S) overnight at 4 °C. The membranes were incubated with horseradish peroxidase (HRP)-conjugated goat anti-mouse IgG antibody (1:5000) as secondary antibody for 1 h at room temperature. For every washing step the membranes were washed for three times for 3 min followed by three times for 10 min with 1 × TBST. Protein bands were detected with chemiluminescence and the Bio-Rad Imager. Protein bands were normalized to total protein after staining the membranes with Coomassie Brilliant Blue-R-250 and de-staining with a mixture containing 10% Acetic-acid and 40% Methanol. Band intensities were quantified with the Bio-Rad’s Image Lab software. Images of the blots of AMPK, AKT1 and PGC1α can be found in figures S1, S2 and S3 respectively.

### Statistics

#### General

All data are expressed as means ± SEM. Statistical analyses were performed using Graphpad Prism 5 (Graphpad Software Inc., La Jolla, California, USA). Differences in daily body mass, body composition and ex vivo measures (F_max_, CT, RT and dF/dt) were tested using a repeated measures two-way ANOVA with group and time as factors. Differences at a specific point were all tested using a two-way ANOVA with tumour and training as factors. All post hoc testing was done with a Bonferroni multiple comparison correction. Differences were considered significant at *p* < *0.05*.

#### PCA + random forest

Principal component analysis (PCA) and random forest analysis were performed using R (R Foundation for Statistical Computing, Vienna, Austria. https://www.R-project.org/). Variables with 2 or fewer missing values were included, missing values were replaced with the mean of the variable. There were 63 variables used for the analyses (Table S2). The package *moments* was used to calculate the Shapiro Wilk p-value, skewness and kurtosis value (Table S2). Random forest analysis was done using the *randomforest* package^39^. For PCA analysis data was auto scaled. Plots were made using the R libraries *gglot2*^40^ and *ggpubr*. Used R scripts are available via https://github.com/mirandavdende/vibrogym-study.

### Microarray gene expression

#### Microarray

RNA from EDL, SOL and heart was isolated (RNeasy Micro kit, Qiagen, Venlo, the Netherlands). Subsequently, RNA was quantified by Nanodrop analysis and integrity was checked by an Agilent 2100 Bioanalyser with RNA 6000 microchips (Agilent Technologies, South Queensferry, UK). Total RNA was labelled with the GeneChip^®^ WT plus Reagent Kit and hybridized to GeneChip^®^ Mouse Gene 1.1 ST Array (Affymetrix, Inc. Santa Clara, Ca, USA). Sample labelling, hybridization to chips and image scanning were performed according to the manufacturers’ instructions.

#### Microarray data analysis

Microarray quality control was performed in MADMAX, a pipeline for statistical analysis of microarray data^41^. Data were normalized using the robust multi-array analysis (RMA) algorithm^42^ as implemented in the Bioconductor package *AffyPLM*. Probe sets were identified with genome information according to Dai et al.^43^ based on annotations provided by the Entrez Gene database, which resulted in the profiling of 22,135 unique genes (custom CDF v22). Differential expression of probe sets (genes) was determined using linear models (package *limma*) and an intensity-based moderated t-statistic^44,45^. T and T + WBV groups were compared to C group for EDL, SOL and Heart samples. P-values were adjusted for multiple testing by Benjamini–Hochberg false discovery rate (FDR) procedure^46^. Probe sets with an adjusted *p*-value of *p* < 0.01 were considered regulated. Venn diagrams and scatter plots were made using the R libraries *gglot2*^40^ and *VennDiagram*^47^. To find genes most regulated by either tumour or training, a sparse Partial Least Squares Discriminant (sPLS-DA) analysis was performed using the *mixOmics* package^48^. Changes in individual genes were related to changes in pathways by gene set enrichment analysis (GSEA)^49^ and the subset of metabolic and signalling pathways retrieved from the expert-curated Kyoto Encyclopedia of Genes and Genomes (KEGG) database^50^. Only gene sets consisting of more than 10 and fewer than 500 genes were considered, which resulted in the inclusion of 226 gene sets. For each comparison, genes were ranked on their t-value that was calculated by the empirical Bayes method. Statistical significance of GSEA results was determined using 10,000 permutations. GSEA and visualization was performed using the Bioconductor package *clusterProfiler *^51^. Microarray data has been submitted to the Gene Expression Omnibus (GEO) database under accession number GSE121972. Used R scripts are available via https://github.com/mirandavdende/vibrogym-study.

### Declarations

This study was conducted in accordance with institutional guidelines for the care and use of laboratory animals established by the Animal Ethics Committee of the University of Wageningen, and all animal procedures related to the purpose of the research were approved under the Ethical license of the national competent authority (registration number 2014075.e), securing full compliance the European Directive 2010/63/EU for the use of animals for scientific purposes. The manuscript does not contain clinical studies or patient data. This research was funded by Wageningen University and VLAG Graduate School. Used R scripts are available via https://github.com/mirandavdende/vibrogym-study. M van der Ende, RLC Plas, M van Dijk, JT Dwarkasing, F van Gemerden, A Sarokhani, HJM Swarts, EM van Schothorst, S Grefte, RF Witkamp and K van Norren give their consent for publication and declare that they have no conflict of interest.

## Results

### No effect of training in tumour bearing mice on general cachectic parameters

**Figure 1:**
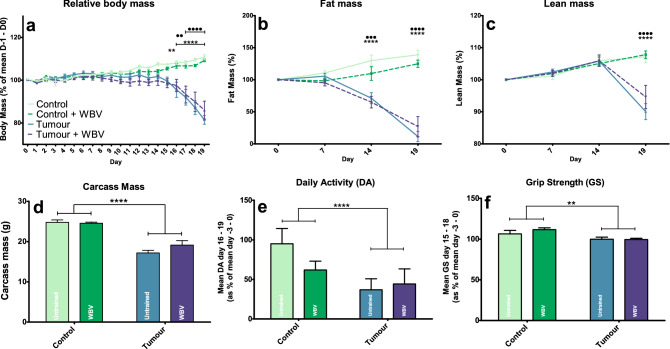
Relative body mass (**a**), fat mass (**b**) and lean mass(**c**) over time, carcass mass at section (**d**), mean daily activity (**e**) and grip strength (**f**) at the end of the experiment. Data represent mean ± SEM. Asterisks indicate significant differences between C and T and dots indicate significant differences between C + WBV and T + WBV. */ •, **/ ••, ***/ ••• and ****/ •••• indicate *p* < *0.05*, *p* < *0.01*, *p* < *0.005* and *p* < *0.001* respectively.

**Figure 2:**
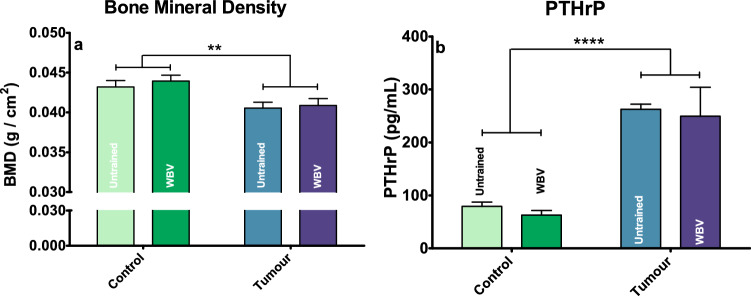
Front leg BMD (**a**) and plasma PTHrP (**b**) at section. Data represent mean ± sem. ** and **** indicate *p* < *0.01* and *p* < *0.001* respectively.

Nineteen days after tumour inoculation, body mass, fat mass and lean mass started to significantly decrease in tumour bearing mice (T and T + WBV) (Fig. 1a–c), with fat mass reduction preceding the other characteristics on day fourteen. Upon sacrifice (day 19), the carcass mass (Fig. 1d) was also significantly lower in the T and T + WBV groups. Almost all organs, muscles (except for SOL) and fat compartments showed decreased mass due to a tumour effect (table S3). However, the mass of the spleen and intestine were both larger in tumour bearing mice, which could be explained by tumour growth and inflammatory reaction. Plasma INFγ and IL-6 showed an increase due to the tumour while the other tested cytokines were not affected (figure S4). Mean grip strength and daily activity at the end of the experiment were both lower in the tumour bearing mice (T and T + WBV) compared to the control mice (C and C + WBV) (Fig. 1e,f). Front leg BMD was significantly lower, but plasma PTHrP was significantly higher in tumour bearing mice (T and T + WBV) compared to control mice (C and C + WBV) (Fig. 2a,b). The gastrocnemius muscle of the T and T + WBV groups showed a significant increase in gene expression of Murf1 and atrogin, which are both markers for muscle mass breakdown (table S3). In all these parameters no effect of WBV was seen in both the C + WBV group and the T + WBV group, with one exception: EDL muscle mass was increased in C + WBV compared to control. Next to that, the C + WBV group showed a trend of a lower mean daily activity, although not significant.

#### Training increases contraction and relaxation speed of SOL, but it has no effect on muscle fibre type

**Figure 3:**
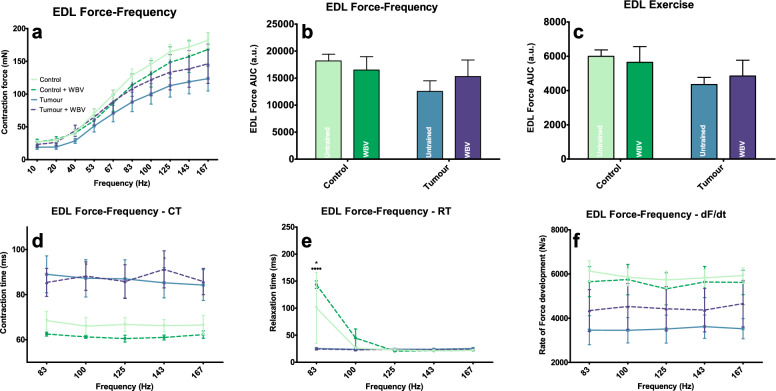
EDL ex-vivo muscle characteristics. Maximal tetanic force, at different stimulation frequencies (**a**) and area under the curve of the force-frequency (FF) relationship (**b**) and the exercise protocol (**c**). Contraction time (**d**), relaxation time (**e**) and rate of force development (**f**). Data represent mean ± SEM. Asterisks indicate significant differences between C and T and dots indicate significant differences between C + WBV and T + WBV. */ • and ****/ •••• indicate *p* < *0.05* and *p* < *0.001* respectively.

**Figure 4:**
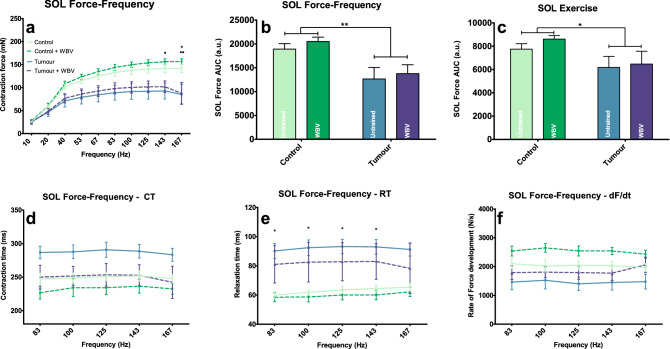
SOL ex-vivo muscle characteristics. Maximal tetanic force, at different stimulation frequencies (**a**) and area under the curve of the force-frequency relationship (**b**) and the exercise protocol (**c**]. Contraction time (**d**), relaxation time (**e**) and rate of force development (**f**). Data represent mean ± sem. Asterisks indicate significant differences between C and T and dots indicate significant differences between C + WBV and T + WBV. */ •, **/ ••and ***/ ••• indicate *p* < *0.05*, *p* < *0.01* and *p* < *0.005* respectively.

EDL only showed minor differences in ex vivo muscle function (Fig. 3a–c,f). Contraction time was slightly longer in the muscle of the tumour mice (T and T + WBV) (Fig. 3d), however, this was not significant. Compared to the C and C + WBV groups respectively, the relaxation time was significantly shorter in the T and T + WBV groups at 83 Hz (Fig. 3e). In all cases, no significant changes due to the WBV training were observed. In the SOL greater differences were seen between the tumour mice (T and T + WBV) and the control mice (C and C + WBV). A significantly lower F_max_ could be observed in both T and T + WBV groups at 167 Hz (Fig. 4a). AUC for both the force-frequency and the exercise protocol was significantly lower in the T and T + WBV groups (Fig. 4b,c). In the rate of force development, no differences were found (Fig. 4f).

In the SOL the clearest training effect was in the contraction and relaxation time. The contraction time of the T + WBV group was similar to that of the C and C + WBV groups while that of the T group seemed slower than in the other groups (Fig. 4d). Also, only the T group showed significantly longer relaxation time compared to the C group, while the C + WBV and T + WBV groups did not significantly differ from each other (Fig. 4e).

**Figure 5:**
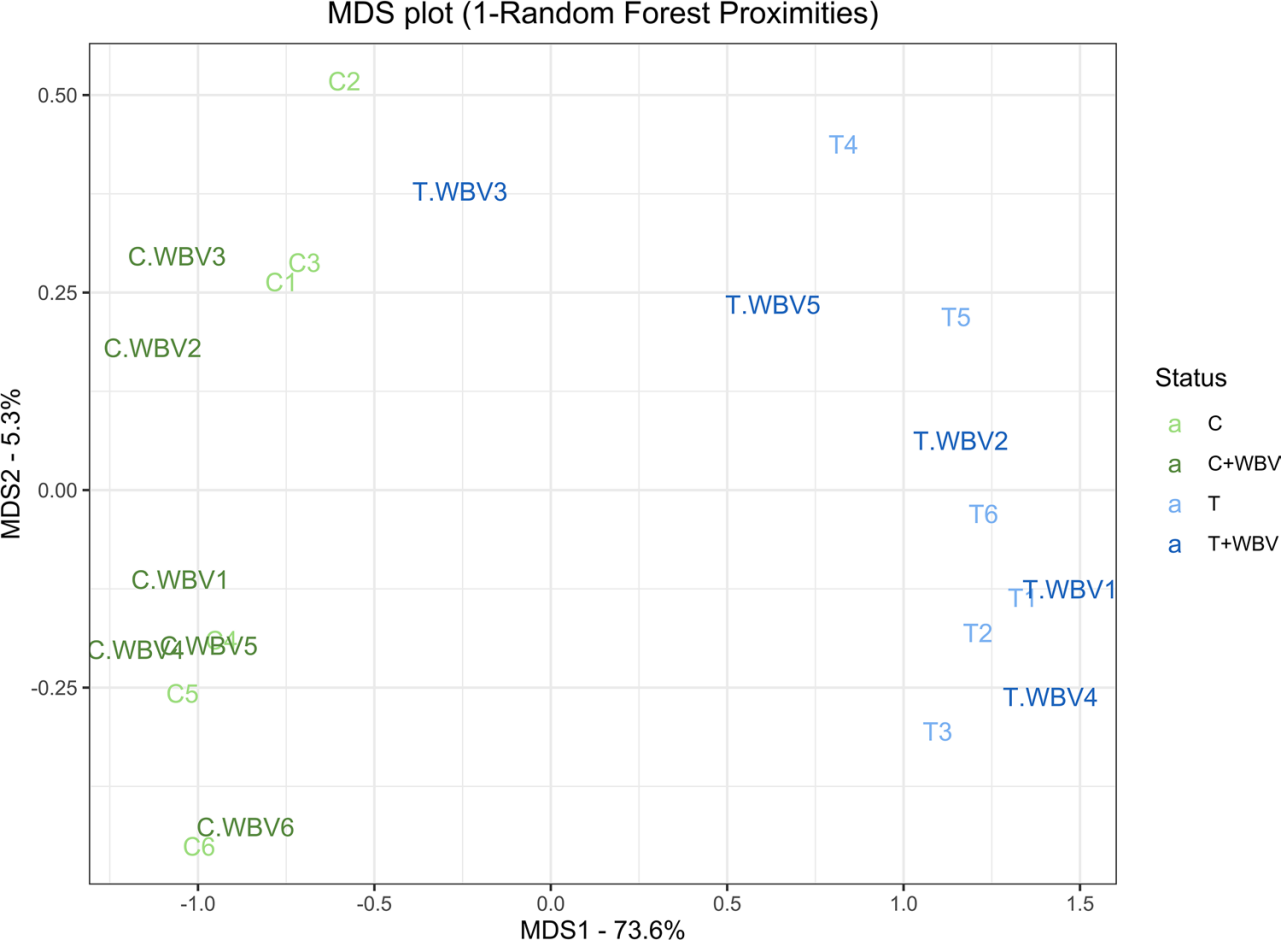
MDS plot (1 – Random Forest Proximities) using 63 variables. Green colour are the control groups and blue colour are the tumour groups, in both the darker colour represents the group with whole body vibration training (WBV).

To further investigate the difference in ex vivo outcomes in the SOL, an immunofluorescent staining to measure fibre properties was performed. No differences between groups were found in total fibre cross sectional area (CSA), type I fibre CSA or type II fibre CSA (Figures S5a–c). A tumour effect in fibre CSA ratio (type II/type I CSA) was found, which was decreased in tumour bearing mice (T and T + WBV) compared to control mice (C and C + WBV) indicating a heavier wasting in type II fibres (Fig. 5d,f).

#### Training in tumour bearing mice lowers AKT1 total protein expression

To assess mitochondrial biogenesis, gene and protein expression levels of *Sirt1*, *Ampk*, *Akt1*, and *Pgc1α* (*Ppargc1α*) were measured in m. gastrocnemius (Table 1). SIRT1, AMPK, and AKT1 are regulators of PGC1α, which is considered the key protein in mitochondrial biogenesis. Sirt1 mRNA expression was upregulated in the T and T + WBV groups. *Ampk* mRNA expression did not change, but total protein expression was significantly lower in the T and T + WBV groups. AKT1, a potentially negative regulator of PGC1α and indirect promotor of protein synthesis, was upregulated at transcript level in the T and T + WBV groups. The mRNA expression level of *Pgc1α* was increased in the tumour bearing mice (T and T + WBV). However, protein expression levels of PGC1α were not significantly changed. Interestingly, total protein expression of AKT1 was significantly decreased by WBV training in the tumour mice (T + WBV), bringing it down to the level of the control group (C).

**Table 1:** Relative gene and protein expression in m. gastrocnemius (mean ± SEM).

Expression	C	C+WBV	T	T+WBV	Statistics	
Pgc1α mRNA	1 ± 0.33	1.27 ± 0.28	3.34 ± 0.99	2.22 ± 0.25	*	Tumour
PGC1α Protein	1 ± 0.1	0.94 ± 0.18	0.79 ± 0.08	0.9 ± 0.1		
P-PGC1α Protein	1 ± 0.09	1.69 ± 0.63	0.62 ± 0.23	0.83 ± 0.27		
P-PGC1 α/PGC1α Protein	1 ± 0.09	2.05 ± 1.07	0.95 ± 0.39	0.85 ± 0.22		
Akt1 mRNA	1 ± 0.12	0.9 ± 0.08	4.04 ± 0.94	3.5 ± 0.58	****	Tumour
AKT1 Protein	1 ± 0.12	0.8 ± 0.13	1.34 ± 0.1	0.91 ± 0.11	*	Training
P-AKT1 Protein	1 ± 0.22	0.51 ± 0.11	1.16 ± 0.32	1.2 ± 0.63		
P-AKT1/AKT1 Protein	1 ± 0.13	64 ± 0.1	0.83 ± 0.18	1.39 ± 0.63		
Ampk mRNA	1 ± 0.14	1.06 ± 0.12	0.85 ± 0.12	1.04 ± 0.09		
AMPK Protein	1 ± 0.11	0.91 ± 0.15	0.27 ± 0.05	0.58 ± 0.17	***	Tumour
P-AMPK Protein	1 ± 0.51	0.64 ± 0.06	1.2 ± 0.54	0.62 ± 0.16		
P-AMPK/AMPK Protein	1 ± 0.44	0.88 ± 0.25	7.99 ± 5.6	1.24 ± 0.27		
Sirt1 mRNA	1 ± 0.2	1.18 ± 0.07	2.16 ± 0.24	1.93 ± 0.19	****	Tumour

Two-way ANOVA with factors Tumour, Training and Tumour–Training interaction and Bonferroni Post-Hoc analysis. Significant effects are represented with * *p* < *0.05*, ** *p* < *0.01*, *** *p* < *0.005*, and **** *p* < *0.001*.

#### No training effect observed when using advanced analysis methods

Next, a random forest and PCA analysis was performed on a combined dataset of all results described above which consists of 63 variables (Table S2). The random forest test is an ensemble learning method for classification, regression and other tasks that operates by constructing a multitude of decision trees. With this approach it is aimed to predict to which group the mice belong (C, C + WBV, T, or T + WBV). The Out of Bag (OOB) estimate of error rate is 73.91%, this means that 73.91% was not correctly classified by the random forest approach. The control mice (C and C + WBV) were not uniquely and correctly classified but rather dispersed in each other’s category which happened for the tumour mice (T and T + WBV) as well. This is also seen in the MDS plot (Fig. 5) in which these two distinct groups can be seen: tumour bearing mice versus the control mice, without any effect of WBV training.

**Figure 6:**
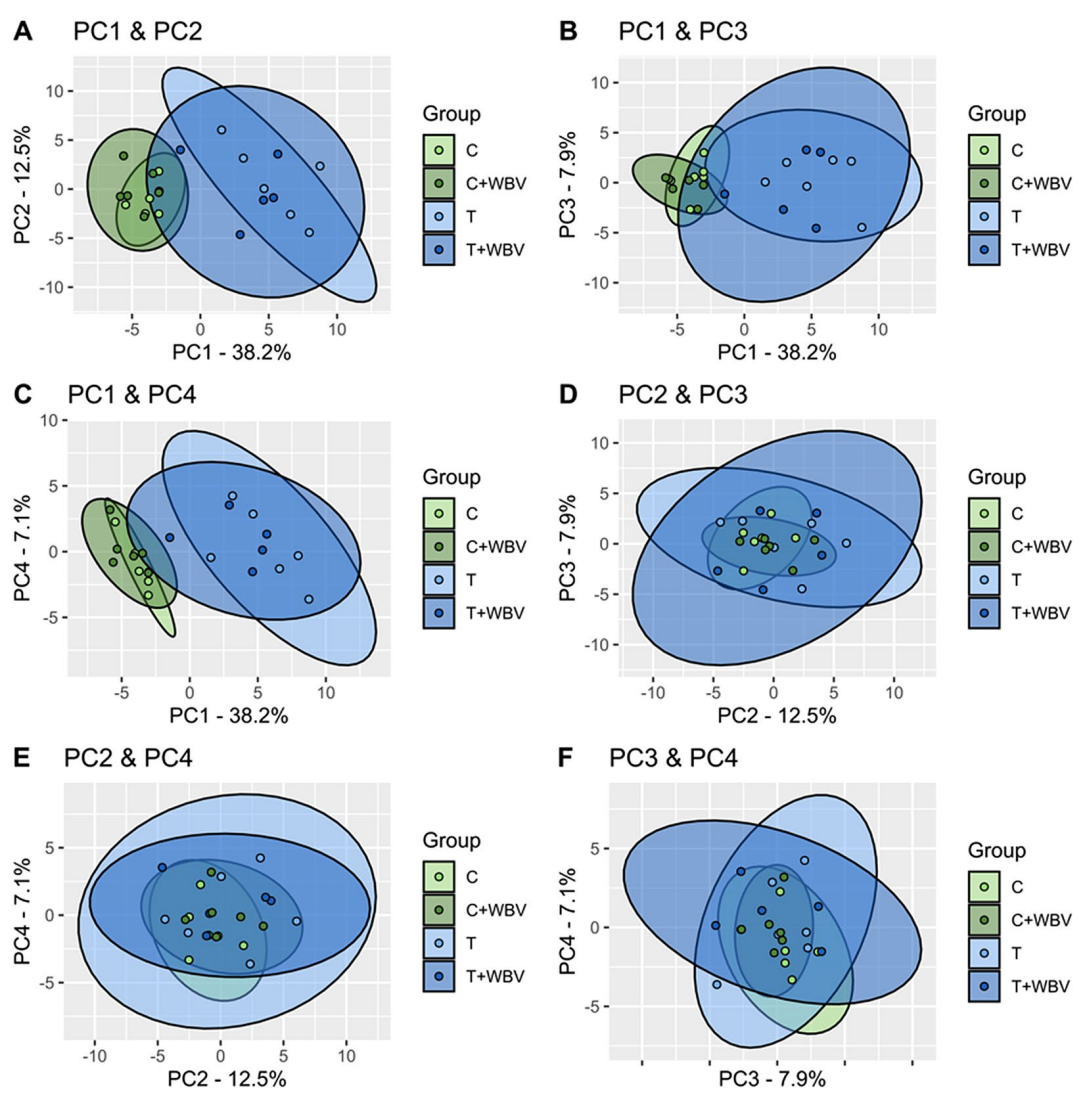
PCA analysis with 63 variables plotting the first four principle components against each other. Green colour are the control groups and blue colour are the tumour groups, in both the darker colour represents the group with whole body vibration training (WBV). PC1 vs PC2 (**A**), PC1 vs PC3 (**B**), PC1 vs PC4 (**C**), PC2 vs PC3 (**D**), PC2 vs PC4 (**E**) and PC3 vs PC4 (**F**).

Next, the scree plot of the PCA analysis (Figure S6) showed that principal component (PC) 1 explains 41% of the variance in this model. When PC1, PC2, PC3, and PC4 were all plotted against each other (Fig. 6) it is clear that only in the plots in which PC1 is involved there is a distinction between tumour bearing mice (T and T + WBV) and control mice (C and C + WBV) (Fig. 6a–c), again without effect of WBV training. When investigating the components in PC1 it was found that carcass weight, body mass, muscle mass, and body composition expressed as lean and fat mass, are the top 5 components. PC2, PC3 and PC4 are not able to make this separation between the groups (Fig. 6d–f).

#### Training attenuates tumour-induced effects on gene expression level in muscle tissue

The physiological experiments described above showed mainly a tumour effect (T and T + WBV). The effects of the whole-body vibration training were mild and only seen in soleus muscle to increase the contraction and relaxation speed and in gastrocnemius muscle to decrease the AKT1 total protein expression. These findings were not enough to make a distinction between the WBV trained and the non-trained mice using random forest analysis or PCA analysis. However, these minor changes contrast with what was observed in the muscle gene expression profiles of these mice and this can provide an indication of how these cells are counteracting the induced effects.

**Figure 7:**
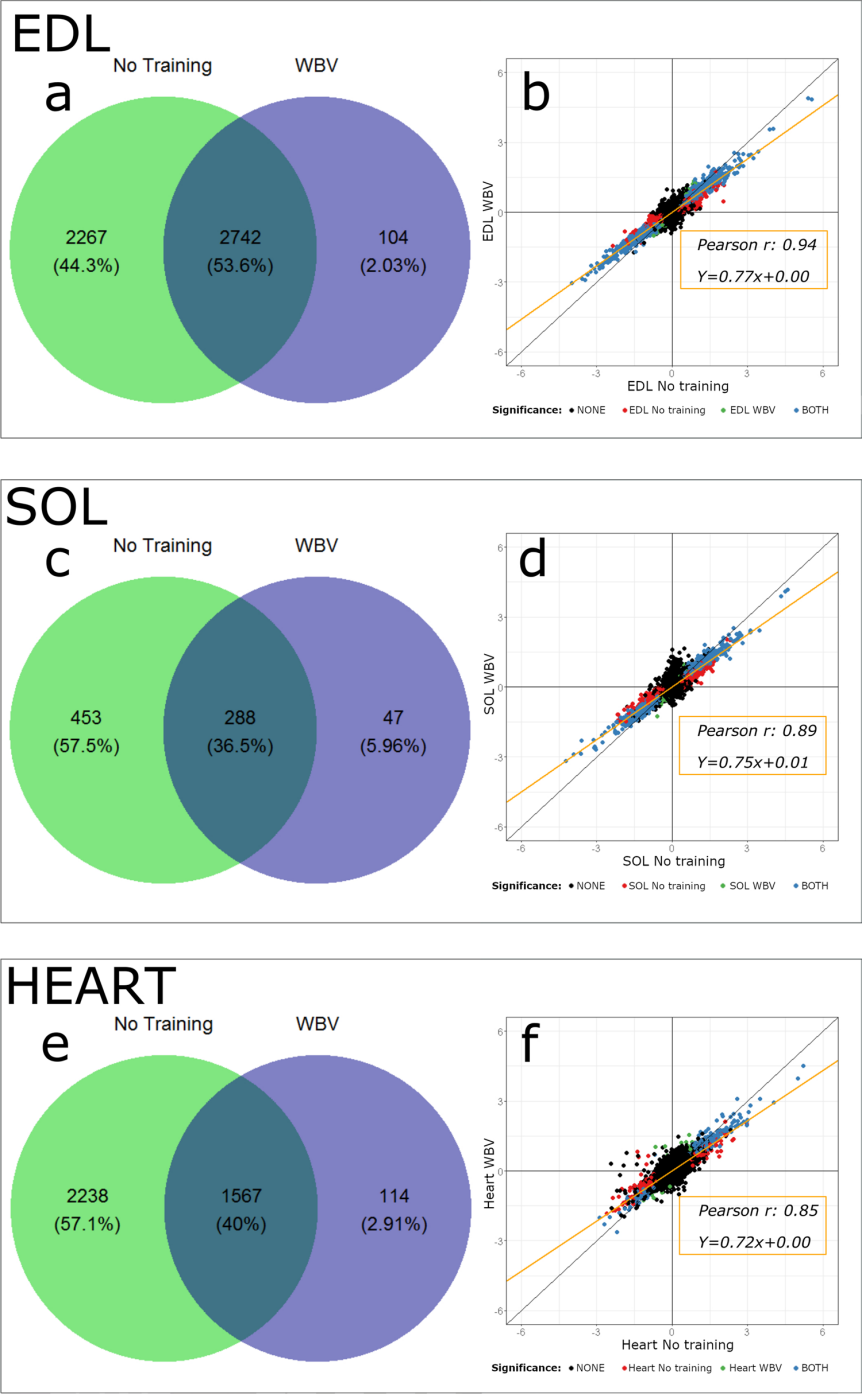
Microarray results showing the comparison of C with T and T + WBV group for three different tissues: EDL (**a,b**), SOL (**c,d**), and heart (**e,f**). Venn diagrams (**a,c,e**) display number of genes with q < 0.01 in T (green) and WBV (blue) mice. Scatter plots (**b,d,f**) show a correlation and linear regression of log-ratio of all genes from T + WBV (y-axis) and T (x-axis) compared to C mice with colors indicating significance.

In both EDL, SOL and heart samples, WBV training reduced the impact of the tumour on the muscle transcriptome. Importantly, fewer genes were significantly changed in the T + WBV group in all muscle tissues than in the T group when comparing each of these groups to the C group (Fig. 7 a,c,e). However, the direction of the change in gene expression was very similar in tumour mice (T and T + WBV) with clear correlations between the log-ratio of all genes (Pearson r of 0.94 [EDL], 0.89 [SOL] and 0.85 [Heart]) Fig. 7b,d,f). Moreover, the magnitude of change in gene expression was similar in all tissues where the slope of a linear regression fit was between 0.72 and 0.77 in all tissues. This means that the genes that significantly changed in both the T + WBV and T groups do this along the same line and to a similar degree.

**Figure 8:**
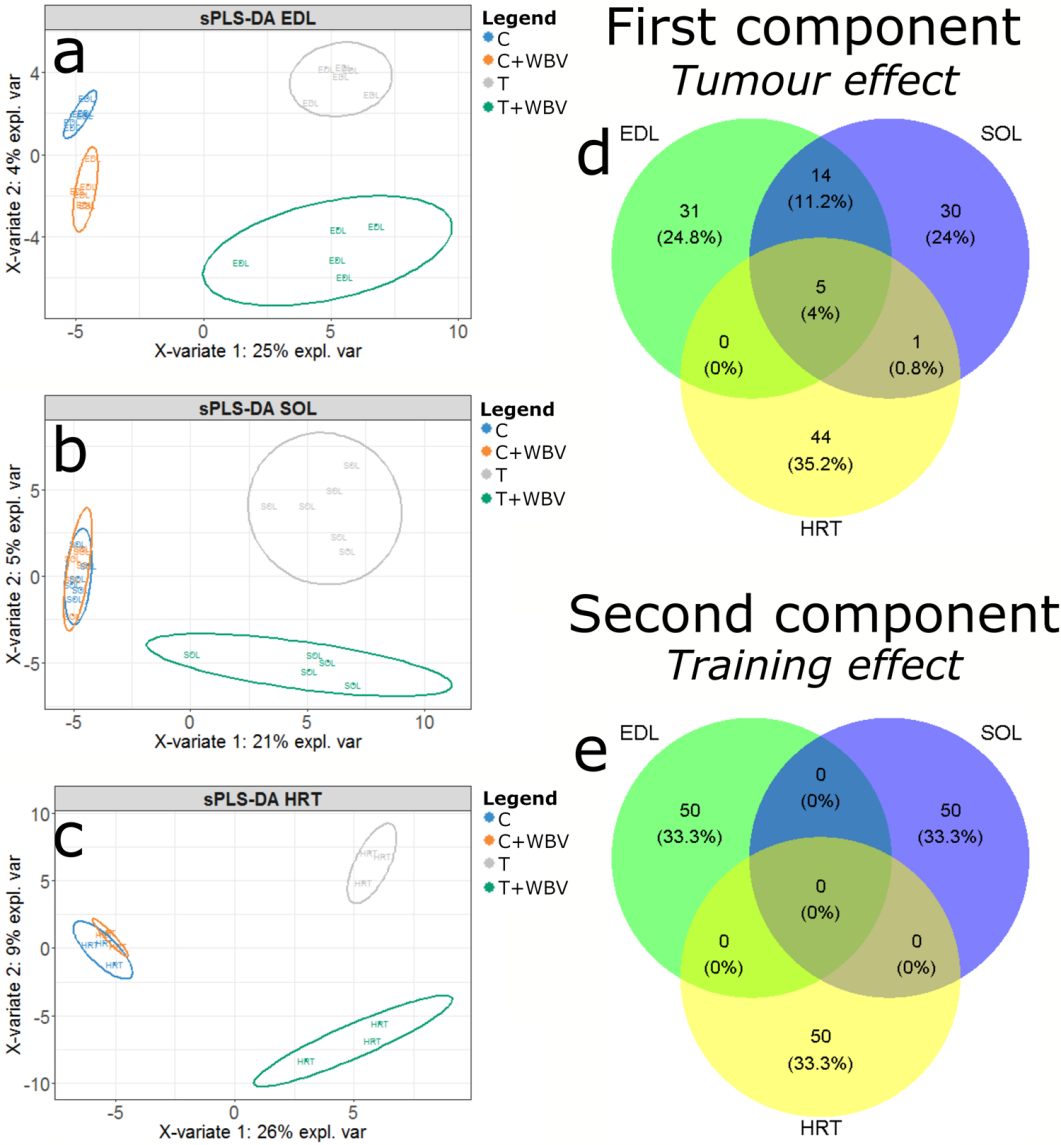
Microarray results showing a partial least squares discriminant analysis of all four groups with two components and 50 genes per component. sPLS-DA is performed per tissue, EDL (**a**), SOL (**b**) and heart [HRT] (**c**). Venn diagrams display genes per tissue in component 1 (**d**) and 2 (**e**).

Subsequently, it was tested if groups could be separated based on gene expression using a sPLS-DA with two components and a maximum of 50 genes per component (Fig. 8). Here it was noticed that the tumour effect (T and T + WBV) was very distinctly explaining 21–26% of variance (Fig. 8a–c) with some common genes in the first component (Fig. 8d). The second component separated groups based on WBV training. For all tissues, tumour bearing mice (T and T + WBV) were separated in trained and untrained where for the control mice (C and C + WBV) only in EDL the groups were separated. This indicates that there was a WBV training effect in the T and T + WBV groups, whereas there was little to no training effect in the C and C + WBV groups on the level of gene expression. The clearest separation based on WBV training was visible in the cardiac tissue with 9% explained variance. No commonalities were found in the genes driving this training effect in the three different muscle tissues (Fig. 8e).

GSEA shows that the proteasome and RNA transport pathways are the most abundantly upregulated pathways for skeletal muscle with no significant regulation in heart muscle in the T and T + WBV groups (figure S7). The complement and coagulation cascades are the most abundantly upregulated pathway in all three muscle tissues, with it being upregulated in the T + WBV group in EDL and heart, while for soleus it was upregulated in both groups (T and T + WBV). While thermogenesis and oxidative phosphorylation pathways are the most abundant pathways being down regulated in EDL, SOL and heart tissue in the T and T + WBV groups. The WBV prevented the upregulation of the proteasome pathway in the SOL of the T + WBV group but not in the EDL.

## Discussion

As it was hypothesized, WBV might be able to attenuate muscle wasting colon-derived-tumour-induced C26 cachexia mouse model. The current study primarily aimed to explore possible modulating effects on cancer-induced loss of muscle mass and function of whole-body vibration training (WBV) in the C26 cachexia mouse model. The cachectic effects found in this model for are in line with those reported in literature; a clear reduction in body, muscle and fat mass of a similar order of magnitude as reported in comparable studies was found^29,52,53^. Moreover, reductions in daily activity^29^, grip strength^54^, BMD^55^, and the increase in muscle *Murf1* and *Atrogin1* expression^56^ are similar to other studies. On cytokine levels a clear IL-6 induction and a small reduction of IFNγ were found, where others also found effects on TNF-α and IL-4^53^. When looking at muscle specific effects similar tumour-induced effects compared to other studies with decreased maximal force and increased muscle contraction and relaxation times were seen^29,57^. Although no significant effect in the EDL was observed, this may result from a lack of statistical power since the data (Figs. 3 and 4) clearly shows a trend in the tumour effect reflected by a decreased maximum force and increasing contraction and relaxation times. For fibre type-specific effects, these results are also in line with other studies concerning the heavier tumour burden on type II fibres compared to that on type I fibres^57^. Taken together, this tumour model largely supports previous findings which led us to conclude that it was suitable to explore effects of WBV on cancer-induced cachexia.

The most interesting finding was that WBV reduced the effects of the tumour on muscle gene expression in EDL, SOL and heart. The WBV prevented the upregulation of the proteasome pathway in the SOL but not in the EDL. Upregulation of the proteasome pathway and the complement and coagulation cascades are also found in another study with the C26 mouse model^58^. In isolated SOL, contractility was improved with contraction and relaxation time of the trained tumour group shifting to, and not being significantly different from, the trained control group. Training decreased AKT1 protein expression in the tumour group (T + WBV). Additionally, WBV effects in the control mice were found (C + WBV). Training increased EDL muscle mass in the control group and, surprisingly, there was a trend for WBV to reduce daily activity in these animals. Taken together, the overall effects of WBV training are small and mainly visible in gene expression, which might represent an adaptive response. The lack of specific effects are likely to be caused by the limited number of animals used, the short period of training or the severity of the cachexia in this study.

Literature on effects of WBV in rodents shows some contradicting results. In the ex vivo set-up of this study an improvement in contractility and a trend of improved strength were found. This is in line with other findings where WBV in healthy mice was shown to partially improve muscle contractility, specifically strength and relaxation rates^24^. Possibly, the reason for the absent effect on muscle morphology, like fibre type and cross-sectional area, could be due to the short period of training in combination with the severity of the cachexia in this study. This is supported by two other studies that also found no significant effect of WBV on myosin heavy chain (MHC) isoforms in the soleus muscle of mice^24,59^. In contrast, a 6-week WBV training in healthy mice did show to increase total cross-sectional area of the soleus muscle as well as its type I and II muscle fibres^26^. A study into the long-term effects of WBV on the gastrocnemius muscle in rats found a decrease in type I content in favour of type II^60^. This could be beneficial for tumour bearing mice as type II fibres tend to be more affected by cachexia^57^. However, this should be further investigated in future studies. In a recent metabolomics study with aged mice it was found that WBV might postpone senility by attenuating lipid deposition and reducing chronic inflammation and the insulin resistance of skeletal muscle^61^. A study comparing aerobic training (treadmill exercise) with resistance training (ladder climbing) in C26 mice indicated possible beneficial effects of aerobic training and not of resistance training^54^. However, aerobic training was only able to mildly preserve muscle size, sensory motor function and relative *m. gastrocnemius* mass but failed to prevent weight loss^54^. This underlines the challenges in the treatment of cachexia with exercise. Another study in Apc^Min/+^ mice showed that moderate treadmill exercise is able to attenuate body and muscle mass loss in IL-6-dependent cachexia, moreover, mitochondrial oxidative capacity was improved^62^. The importance of the mitochondria can also be seen in the GSEA results of the current study, where oxidative phosphorylation in muscle is one of the top enriched and downregulated pathways in the tumour bearing mice (T and T + WBV). The relevance of mitochondrial dysfunction is emergent from literature^63^. Nevertheless, these data support the view that in muscle metabolic adaptations take place induced by the tumour suggesting a switch from oxidative to glycolytic muscle metabolism. Moreover, proper muscle mitochondrial function relies on proper mRNA handling of nuclear encoded mitochondrial genes. Souza et al. showed the importance of mRNAs encoding essential mitochondrial regulators, as their stability is a function of muscle oxidative capacity^64^. Knowing this, one could hypothesize that the increase expression of the RNA transport pathway might be a compensation mechanism to counteract the downregulated oxidative phosphorylation pathway in tumour bearing mice. However, the upregulation in *Pgc1α* gene expression did not result in an higher PGC1α protein expression in the current study.

Unfortunately, the four groups could not fully be separated with PCA or random forest analysis. This could be attributed to the fact that the differences were limited or, alternatively, that the parameters to separate the training mice were not measured as for instance the gene expression profiles were not included in this analysis. This is a legit possibility because clear differences were seen in these profiles and the tumour bearing groups could be separated in trained versus untrained using a sPLS-DA. Taken together, these results and those from previous studies seem to indicate that different forms of WBV training have mostly small but distinct effects. This might indicate that WBV could have additive effect in cachexia treatment, however, it is not powerful enough on its own. One of the reasons might be the absence of a preventive effect of WBV on the downregulation of gene-expression of the oxidative phosphorylation pathway. Therefore, WBV in combination with either nutrition or exercise therapy or a combination of both is worthwhile to investigate. In Lewis lung carcinoma bearing mice, endurance training combined with supplementation of the poly unsaturated fatty acid eicosapentaenoic acid proved to partially rescue muscle strength and mass^65^. Moreover, a specific nutritional combination high in fish oil and leucine was able to reduce cachectic symptoms and improve functional performance and immune function^29,53^. Therefore, it would be tempting to investigate whether combining whole body vibration training with endurance training and targeted nutrition might give synergistic effects. As stated before, cancer cachexia patients can struggle with exercise training^15^. WBV is easy to use and, although the small positive effect of WBV, it could be of added value to the therapy. It could be specifically helpful in a tailor made exercise program in patients with cancer-induced fatigue^66^. It might be worthwhile, to initiate follow-up studies that investigate a multiple-targeted treatment, including exercise and nutrition, or to study whole-body vibration training in a model more closely representing the situation in humans.

## Strengths and limitations

As described in the discussion, the muscle wasting colon-derived-tumour-induced C26 cachexia mouse model used in the current study was similar to cachexia models in literature^29,52–56^. Therefore, one strength of this study is the use of a solid cachectic model. An additional strength is the WBV protocol used, as it has yielded beneficial results in previous rodent studies^24,26,28,30^. A limitation of this study is that the WBV protocol was not as effective as expected in this severe cancer cachexia model. A different WBV protocol could have yielded different results, as frequency and recovery time have a dose-dependent effect^67^. A Delphi consensus study for animals could help to have more consistent form of reporting WBV study protocols^22^. Despite the limitations, the current study has brought cachexia researchers a step closer to possible treatments.

## Conclusion

In conclusion, this data suggest that with the present setup, WBV had only little effect on systemic endpoints but it triggered distinct subtle effects on muscle specific cachexia related pathways in tumour bearing mice. No effects were confirmed on body mass, body composition, inflammation, or bone mineral density, but this could be related to the limited number of animals that were part of this study, the short period of WBV training or the severity of cachexia. Although small, the muscle specific findings are very consistent, and no adverse effects were found. Specifically, the gene-expression data are promising, since far fewer genes significantly change in EDL, SOL and heart muscle of the tumour mice when they had undergone WBV training, showing a gene expression profile that shifts towards control mice. However, the C26 model might have been too acute to study long term effects of a low impact training such as WBV. It is important to publish these minor effects that WBV has in a severe state of cachexia. Additionally, this is the first study looking at the potential beneficial effects of WBV in cachexia. Since the possible effects on different outcomes were examined*,* both in vivo and ex vivo*,* it is possible to pinpoint th*e* level at which vibration training might have an effect, namely an anticipating or adapting gene expression profile.

## Supplementary Information

Supplementary Video S1.

Supplementary Information.

## Abbreviations

AUC: Area under the curve
BMD: Bone mineral density
COPD: Chronic obstructive pulmonary disease
CSA: Cross-sectional area
CT: Contraction time
DEXA: Dual energy X-ray absorptiometry
dF/dt: Rate of change of force
dNGS: Diluted normal goat serum
EDL: M. extensor digitorum longus
ELISA: Enzyme-linked immunosorbent assay
FDR: False discovery rate
FF: Force frequency
F_max_: Maximal force
GEO: Gene expression omnibus
GSEA: Gene set enrichment analysis
HBSS: Hanks’ balanced salt solution
KEGG: Kyoto encyclopedia of genes and genomes
MHC: Myosin heavy chain
PBS: Phosphate buffered saline
PC: Principal component
PCA: Principal component analysis
RMA: Robust multi-array analysis
RT: Relaxation time
SOL: M. soleus
sPLS-DA: Sparse partial least squares discriminant
WBV: Whole body vibration
WGA: Wheat germ agglutinin
