# Supplementary material for: Effects of whole-body vibration training in a cachectic C26 mouse model
Source: Sci Rep. 2021 Nov 3;11:21563. doi: 10.1038/s41598-021-98665-7 (PMC8566567; doi:10.1038/s41598-021-98665-7)
Supplement: Supplementary file 2 — Supplementary Information. [file 41598_2021_98665_MOESM2_ESM.docx]

# Supplementary Information

## Figures


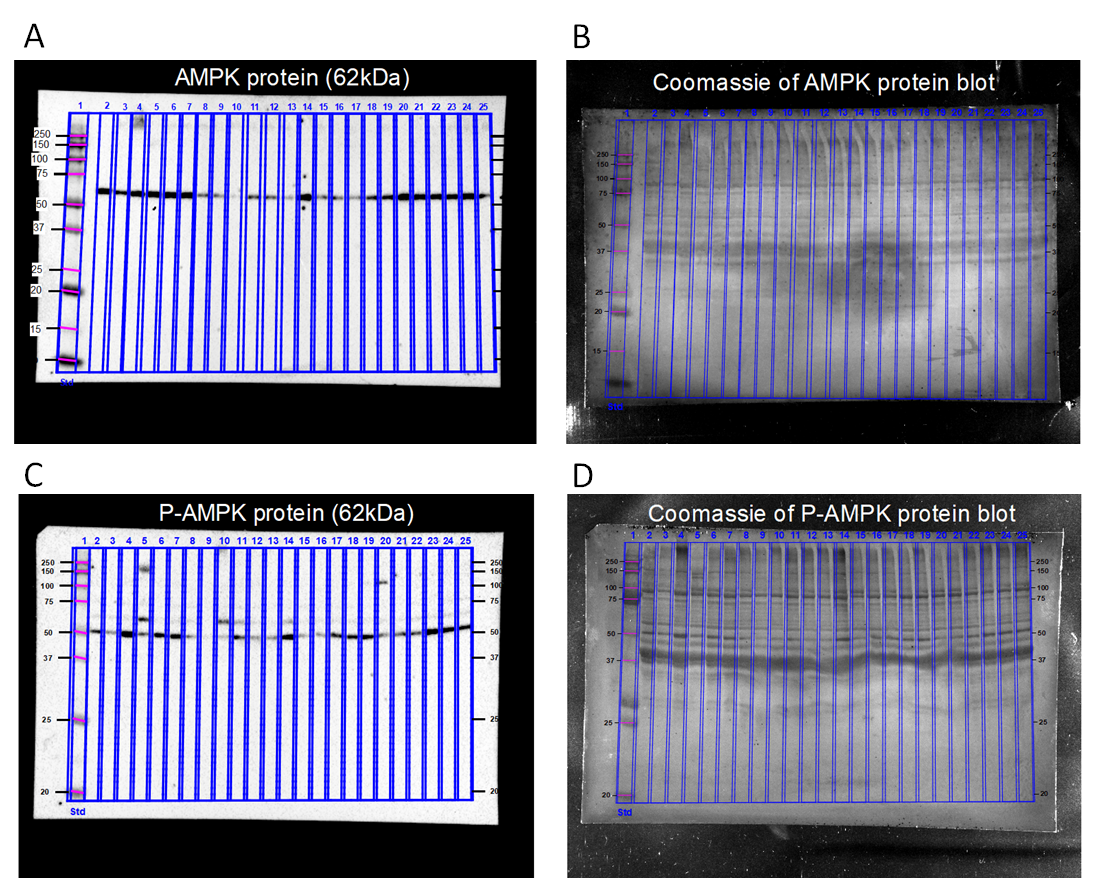


**Fig. S1** Western blot images of AMPK protein [A] plus coomassie staining of the same blot [B] and phopho- AMPK protein [C] plus coomassie staining of the same blot [D]. Marker is indicated on the left side of the blot.


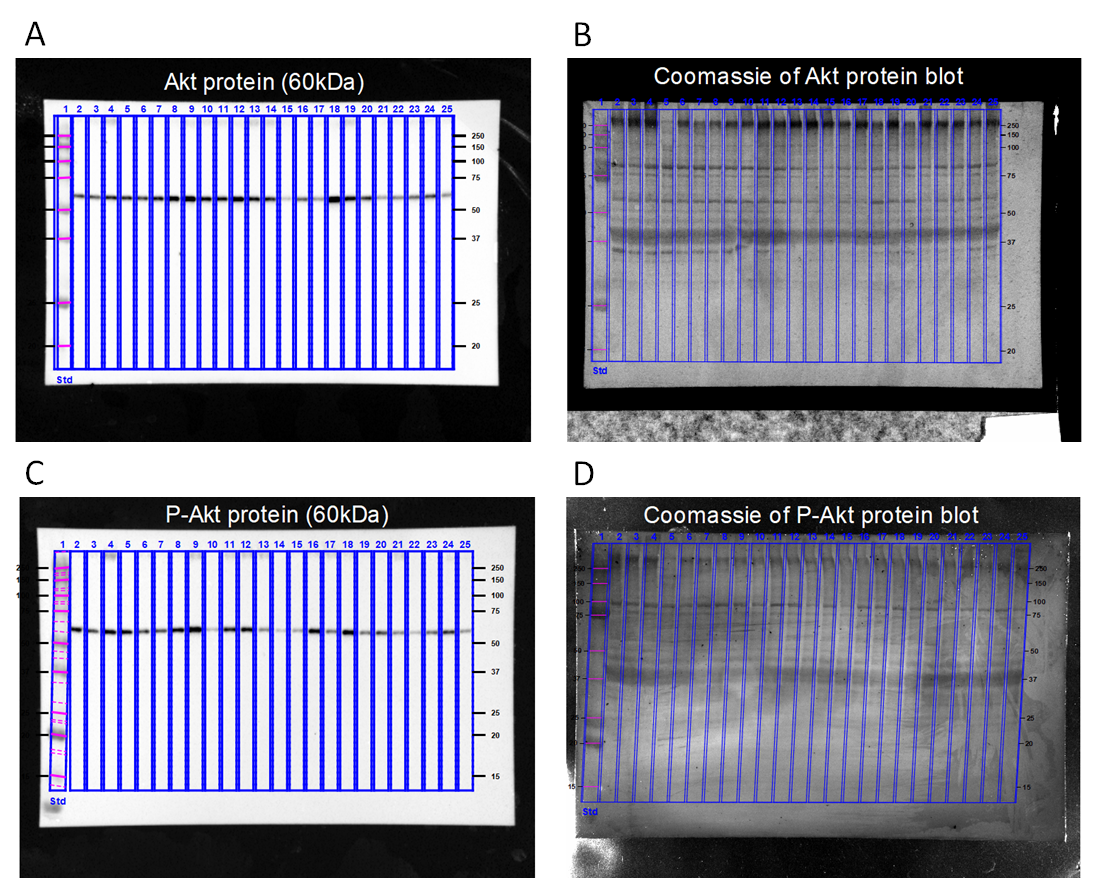


**Fig. S2** Western blot images of Akt protein [A] plus coomassie staining of the same blot [B] and phopho-Akt protein [C] plus coomassie staining of the same blot [D]. Marker is indicated on the left side of the blot.


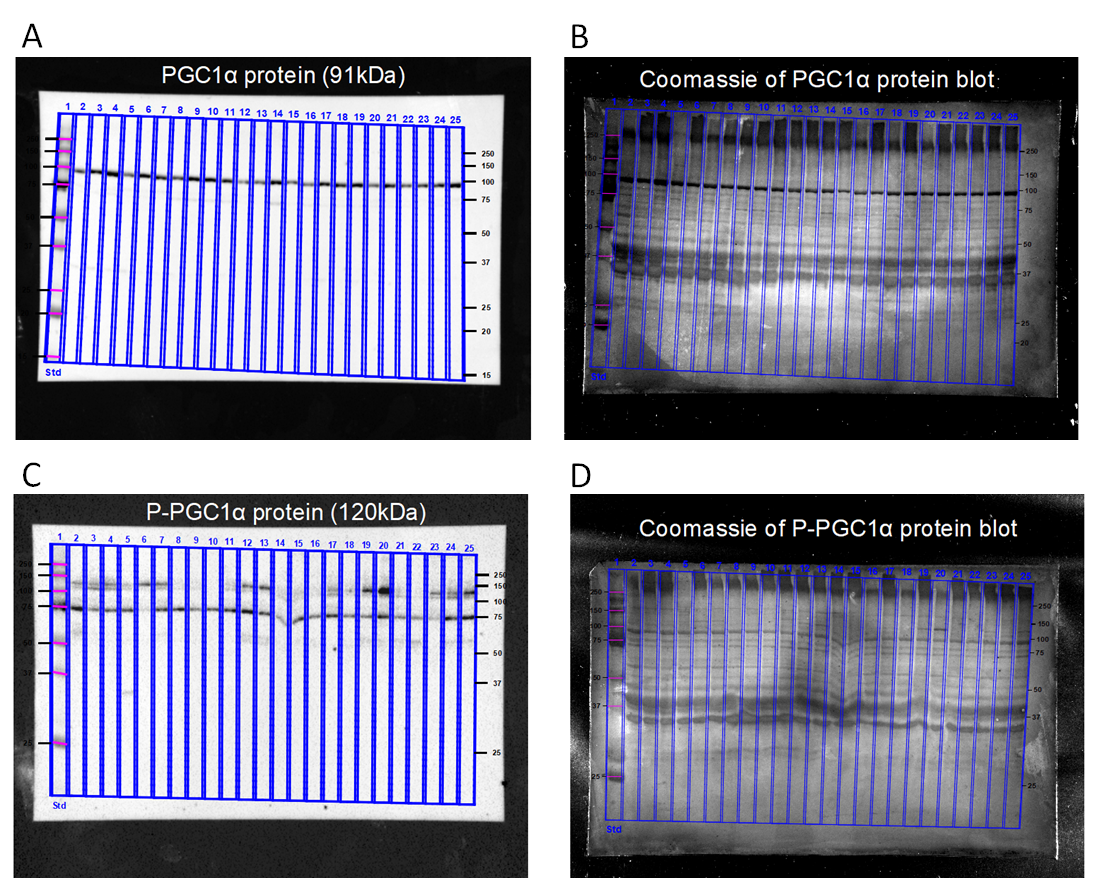


**Fig. S3** Western blot images of PGC1α protein [A] plus coomassie staining of the same blot [B] and phopho- PGC1α protein [C] plus coomassie staining of the same blot [D]. Marker is indicated on the left side of the blot.


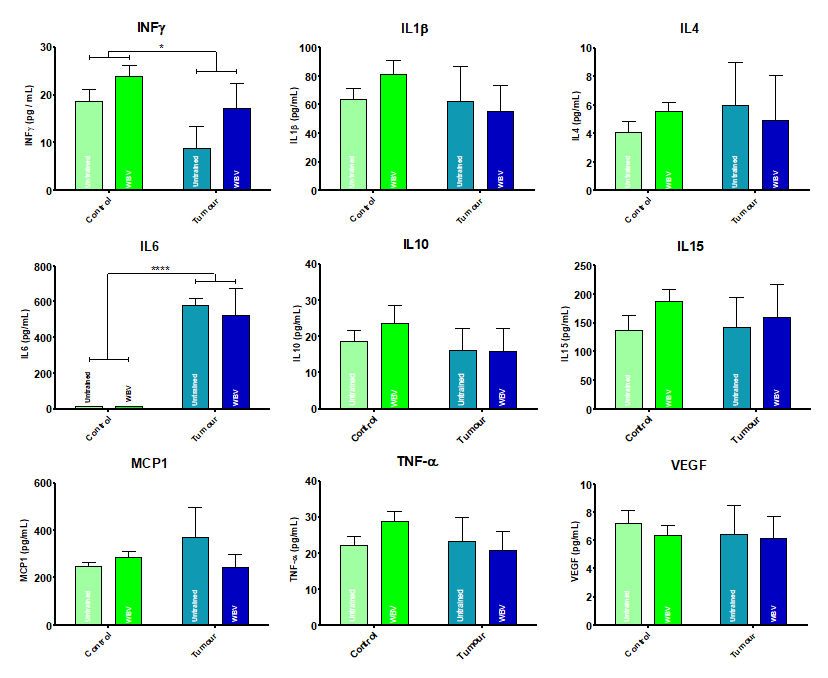


**Fig. S4** Plasma cytokine levels. Data represent mean ± sem. * and **** indicate *p<0.05* and *p<0.001* respectively.


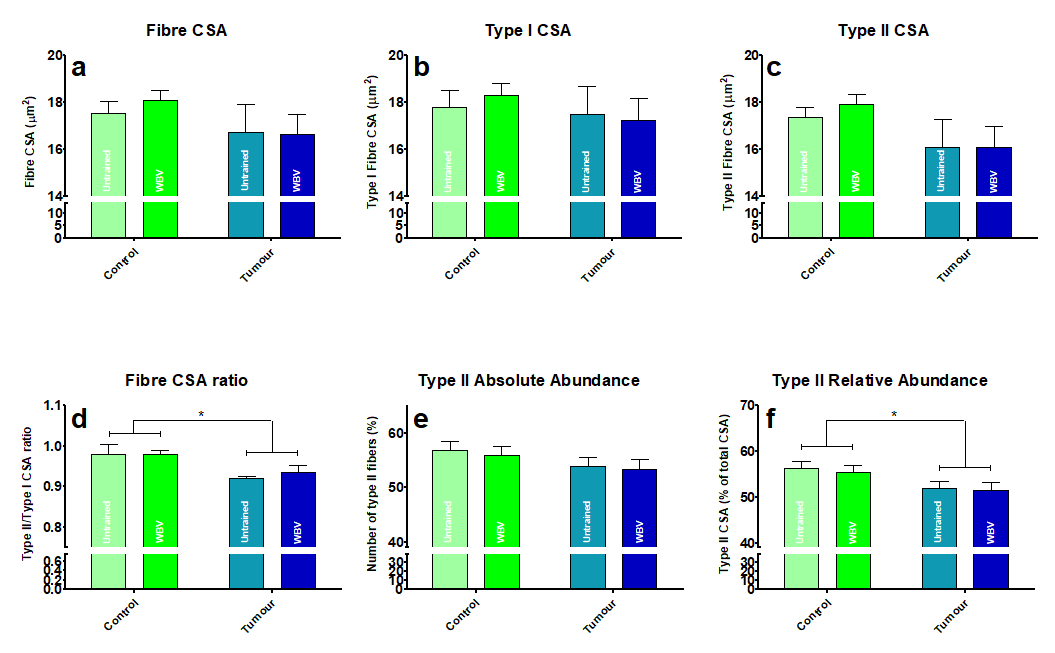


**Fig. S5** SOL muscle fibre characteristics. Fibre cross sectional area of all fibres [a], type I dominant fibres [b] and type II dominant fibres [c]. Relative measures for fibre CSA ratio [d], type II dominant fibre abundance (in % of total fibres) [e] and type II dominant relative abundance (sum of total type II CSA as % of sum all fibre CSA) [f]. Data represent mean ± sem. * indicates *p<0.05*.


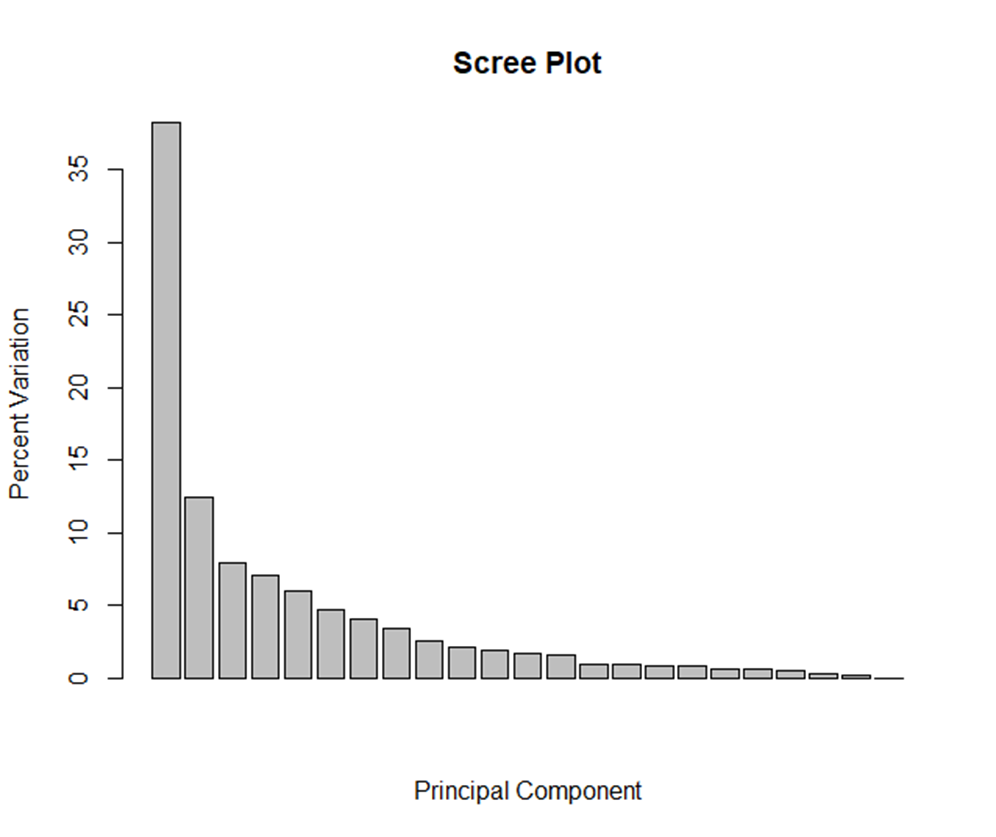


**Fig, S6** Scree plot plotting the principle components of the analysis with 63 variables.


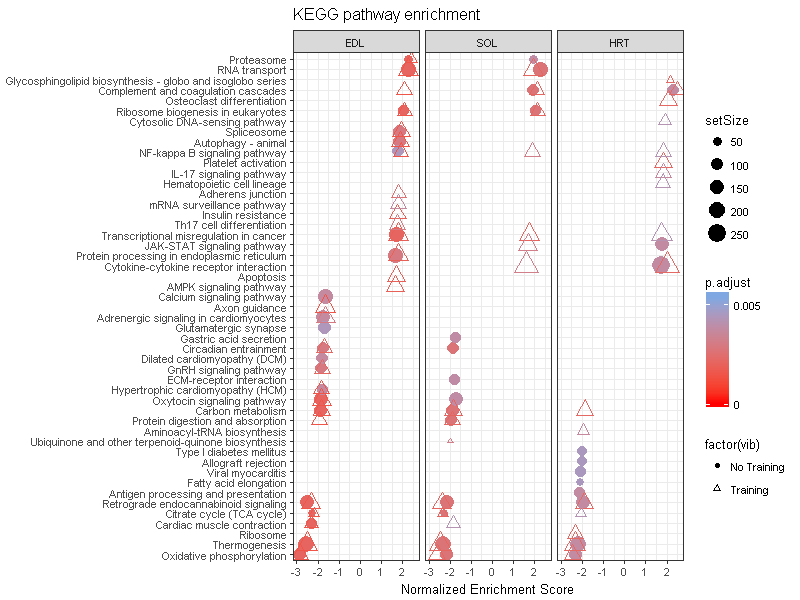


**Fig. S7** Microarray results showing enrichment of KEGG pathways of tumour groups (with and without WBV) compared to control group without WBV.

## Tables

**Table S1** Primer sequences.

**
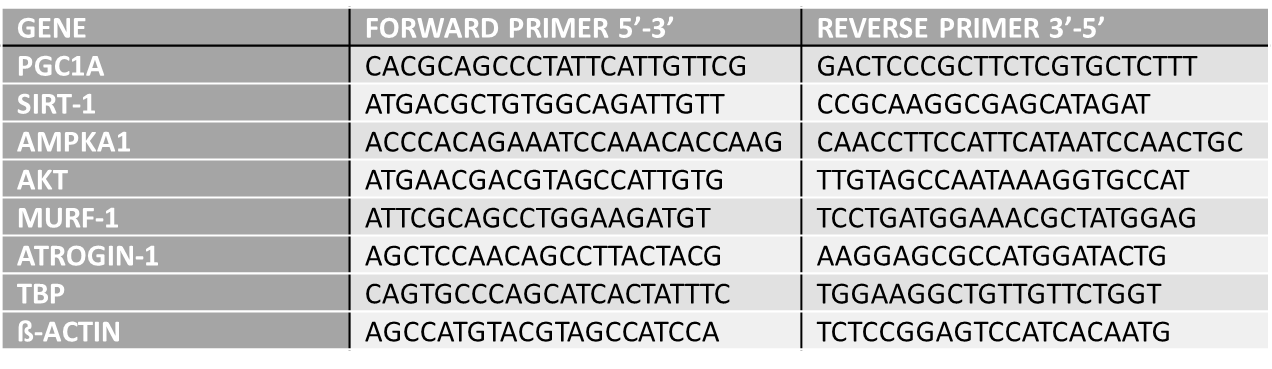
**

**Table S2** Shapiro Wilk p-value, skewness and kurtosis value of the 63 variables used to build the random forest and PCA models.


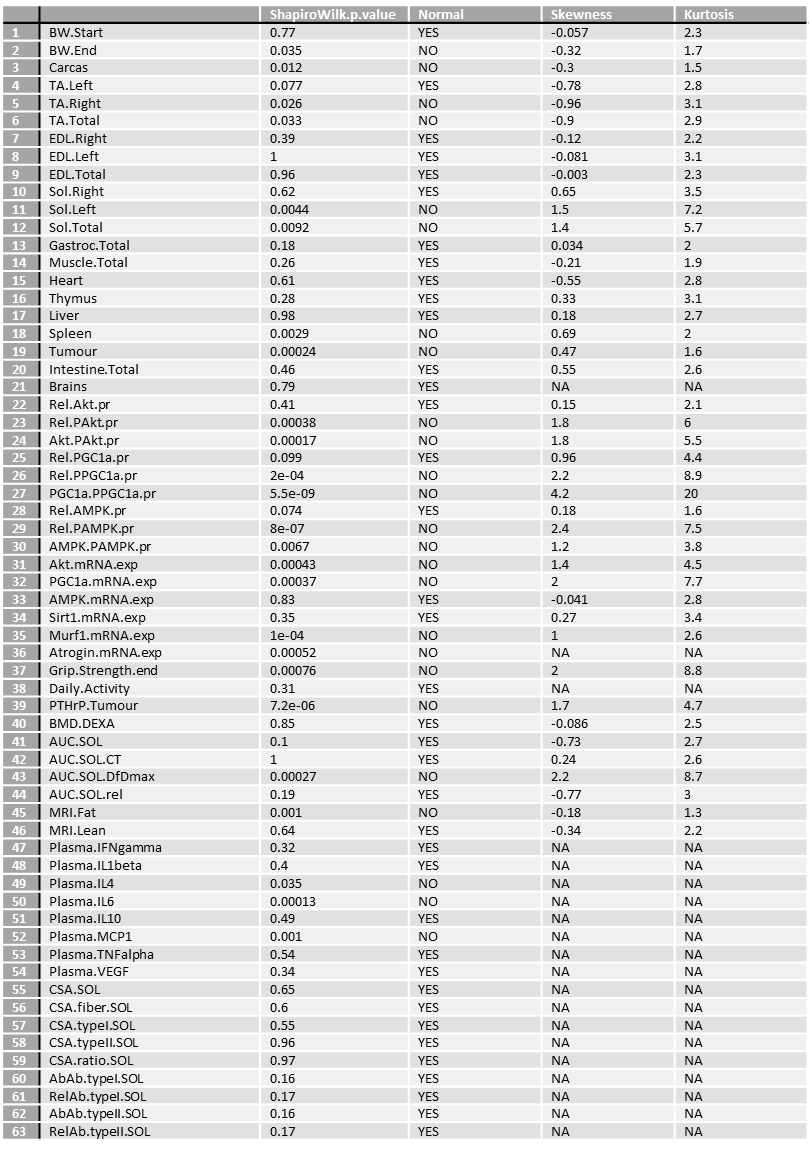


**Table S3** Organ masses (mg) and relative gene expression at section (mean ±SEM). Two-way ANOVA with factors Tumour, Training and Tumour-Training interaction and Bonferroni Post-Hoc analysis. Significant effects are represented with * *p<0.05*, ** *p<0.01*, *** *p<0.005*, and **** *p<0.001*.


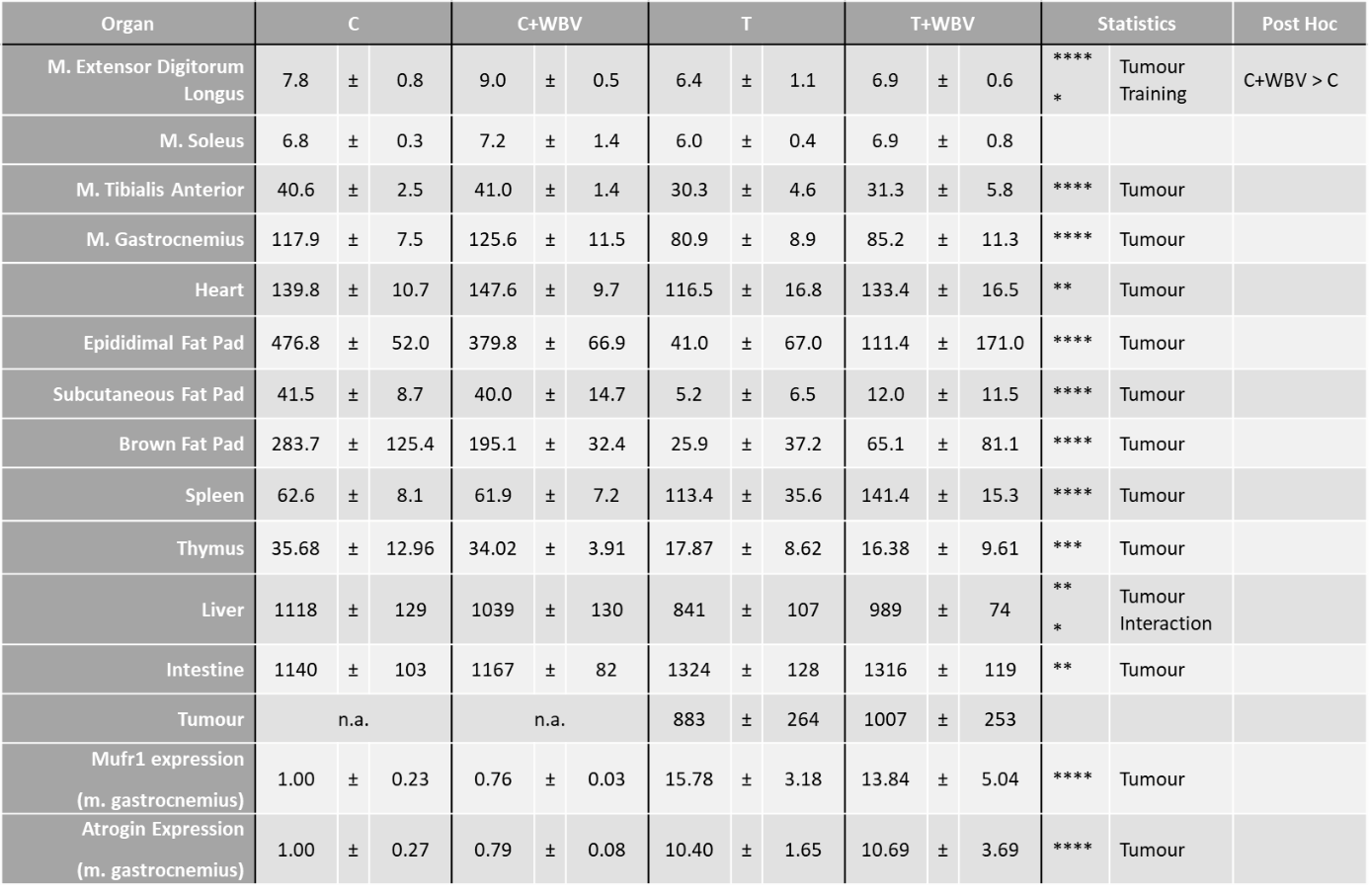


## Video


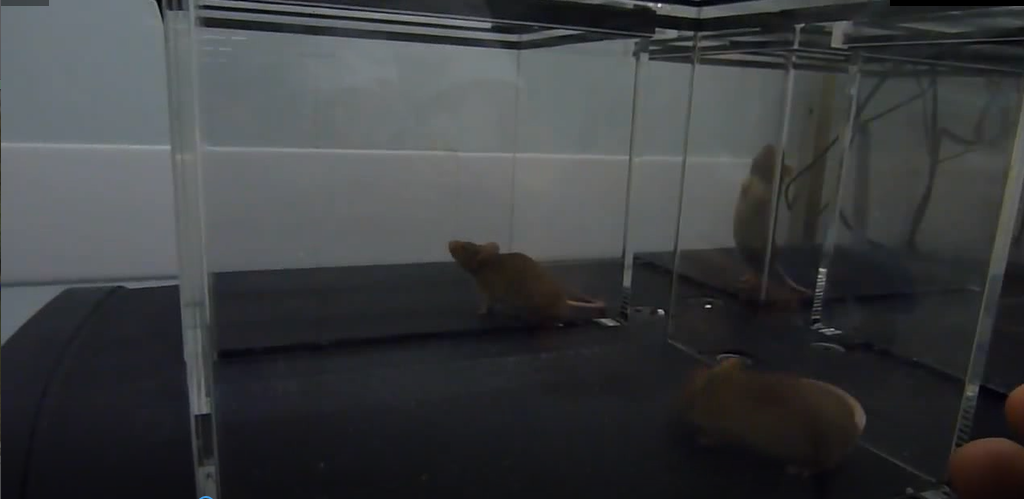


**Video S1** Short clip showing the reaction of the mice to the start of the vibration training. When the stopwatch is started the vibration plate is started and all mice in the video are subjected to the vibration.
